# Leaving Home

**Published:** 1870-12

**Authors:** 


### LEAVING HOME.

When a child leaves home for the first time, after having the parental eye to watch over every footstep and guard against every danger and harm; when for the first time that ever present, ceaseless, sleepless, affectionate care has been withdrawn, whether that absence is to be for a day, or week, or month, or years, there is a painful anxiety on the part of the parent, to give such counsels as may meet the circumstances which are most likely to present themselves. A physician's mind, deeply impressed with the frail tenure of human life, knowing the trifling circumstances which frequently put an apparently healthy child in the grave in a few days, labors, not to tell all that may be requisite to insure a safe return — for that would burden the memory, confuse the mind, and be soon forgotten, — but aims to present some few important points, some wide reaching, general principles, which may impress themselves on the memory of the youngest and most thoughtless.

The thing which may quickest kill, is eating a hearty supper at the close of the first day's travel. While the journey is sure to induce a ravenous appetite, the bodily exercise and mental excitement will leave both mind and body in a state of depression, debility, and exhaustion; while the thought of being from home, away from father and mother, and friends and kindred, and among strangers and strange scenes, so weighs upon the spirits, all combined, leave a person precisely in that condition least capable of resisting even slight causes of disease. Hence if the stomach is overloaded, and a cold close room should be occupied; or an unused bed or damp sheets, an attack of bilious colic, wasting diarrhœa, convulsions, or fatal diarrhœa may very easily destroy life in twenty-four hours. Hence, especially when from home and as a general habit of life —

1. At supper take nothing but a single cup of hot drink, some cold bread and butter, and nothing else.
2. Eat only at regular meal-times.
3. Cut your meat in very small pieces.
4. Eat slowly.
5. Give instant attention to nature's calls, always.
6. Go to a warm place, and avoid having the wind blow on you after exercise, play, or a walk.
7. Remove promptly all damp garments; never stand or sit a moment in a damp place, or sleep near an open door or window.

